# Delayed femoral vein ligation reduces operative time and blood loss during hip disarticulation in patients with extremity tumors

**DOI:** 10.1515/med-2021-0372

**Published:** 2022-10-27

**Authors:** Hongwei Yu, ShuHuai Wang, Qi Song, Yan You, Junjie Bao, Meng Yao

**Affiliations:** Department of Orthopaedics, The Tumor Hospital Affiliated to Harbin Medical University, Harbin, Heilongjiang, China; Department of Pathology, The Tumor Hospital Affiliated to Harbin Medical University, Harbin, Heilongjiang, China; Department of Orthopaedics, The Hospital of Orthopaedics and Traumatology in Harbin, Harbin, Heilongjiang, China; Dermatology Department, The Fourth Affiliated Hospital of Harbin Medical University, Harbin, Heilongjiang, China; Department of Orthopaedics, The Second Affiliated Hospital of Harbin Medical University, No. 246, Xuefu Road, Nangang District, Harbin 150001, Heilongjiang, China

**Keywords:** hip disarticulation, delayed femoral vein ligation, operation time, blood loss

## Abstract

This study aimed to evaluate the effects of delayed femoral vein ligation on the clinical outcomes of hip disarticulation. We retrospectively reviewed 20 patients with extremity tumors (10 bone tumors and 10 soft tissue sarcomas [STS]) who underwent hip disarticulation. Patients treated for hip disarticulation with synchronous femoral vein ligation (*n* = 10, regular surgery group) and hip disarticulation with delayed femoral vein ligation (*n* = 10, delayed ligation group), respectively, were enrolled in this study. The operative time and blood loss were used to evaluate the clinical outcomes. The delayed ligation group had significantly lower operative times than the regular surgery group (*P* < 0.05). Total, hidden, and intraoperative blood loss were all significantly lower in the delayed ligation group than in the regular surgery group (*P* < 0.05). However, there were no significant differences in postoperative blood loss. In conclusion, delayed femoral vein ligation could significantly reduce the operative time, hidden blood loss, and intraoperative blood loss in patients undergoing hip disarticulation.

## 1 Introduction

Sarcomas are rare malignant mesenchymal tumors that constitute approximately 1% of all malignancies. Most osseous and STS are now managed with limb salvage surgery, such as internal pelvic resection [1,2]. However, in circumstances of advanced disease, wherein limb salvage surgery affects the ability to obtain a clear edge during resection, hindquarter amputation may be considered.

Amputation has been extensively used in orthopedic clinics, mostly when the limbs cannot be preserved owing to various factors, such as trauma, infection, tumors, or vascular disease [3]. Amputations include hemipelvectomy, hip disarticulation, and femur amputation (upper 1/3), commonly used for treating extremity tumors [4,5,6].

Most amputations caused by distal limb diseases can be performed in the hemostatic band [7,8]. Tourniquets could not be used in the amputation of the proximal limb. For some diseases, the blood supply at the distal end of the limb is rich, and the effective blood volume is large, especially in the processes of hemipelvic, hip-joint, and mid-upper 1/3 femur amputations. Owing to the large incision wound and the average amount of bleeding (dominant blood loss), amputated limbs take away a large amount of normal blood (hidden blood loss) [9]. Patients had increased total blood loss and significantly decreased hemoglobin after the operation. These conditions increased the probability of blood transfusion in the perioperative period, increased the risk of the perioperative period, and prolonged the treatment period [10]. Therefore, effective hemostasis methods are urgently needed during hip disarticulation. There are various hemostatic methods to reduce intraoperative blood loss during amputation. Effective hemostasis could reduce the perioperative risk and reduce the operative time and recovery period after surgery [11]. Preoperative embolization can reportedly decrease intraoperative blood loss significantly and facilitate the maximal removal of giant-cell tumors [12–16]. Although these methods can reduce blood loss during amputation, there is still a need for the improved hemostasis technique.

In this study, we aimed to apply delayed ligation of the main limb vein in amputated patients and evaluate the curative effect from the aspects of operation time and blood loss. Our findings may provide an effective hemostasis method for hip disarticulation, which had positive clinical benefits.

## 2 Methods

### 2.1 Subjects

**Figure 1:**
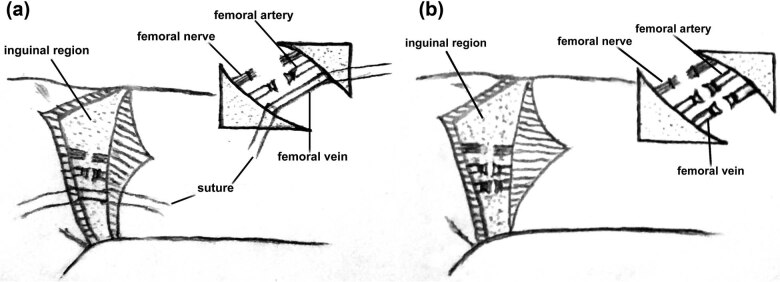
Illustration of (a) hip disarticulation with synchronous ligation of the femoral artery and vein and (b) hip disarticulation with delayed femoral vein ligation.

Twenty patients (14 males and 6 females) with extremity tumors (10 with bone tumors and 10 with STS) were retrospectively reviewed from the Department of Orthopedics in the Tumor Hospital Affiliated to Harbin Medical University from December 2013 to February 2016. The study enrolled hip disarticulation patients treated with synchronous femoral vein ligation (*n* = 10, regular surgery group, Figure 1a) and hip disarticulation with delayed femoral vein ligation (*n* = 10, delayed ligation group, Figure 1b).

Inclusion criteria: (1) The patient ages were in the range of 10–60 years and weighted 35–90 kg, (2) patients suffered from primary malignant limb tumors without limb preservation and had preoperative hemoglobin levels >10 g.

Exclusion criteria: The patients diagnosed with primary or secondary coagulation dysfunction, patients who required massive preoperative blood transfusions, long-term oral anticoagulant therapy patients, or those who underwent interventional therapy for vascular emboli of the affected limbs, and excessive pressure fluctuations during operation, were excluded.

The same experienced surgeons performed the two hip disarticulation procedures. The patients did not receive hemostatic or anticoagulant drugs intraoperatively, and the preoperative hemoglobin was greater than 10 g/dL. Table 1 shows demographic and clinical data.

**Table 1:** Demographic and clinical characteristics of patients treated with regular surgery or delayed ligation hip disarticulation

	Regular surgery group (*n* = 10)	Delayed ligation group (*n* = 10)	*P*-value
Age (year)	28 ± 13	29 ± 15	0.745
Gender (male/female)	7/3	7/3	1.000
Height (cm)	165 ± 10	166 ± 9	0.796
Weight (kg)	63 ± 18	59 ± 11	0.583
Amputated side (left/right)	5/5	4/6	—
Tumor classification (bone tumor/STS)	4/6	6/4	—
Preoperative hemoglobin (g/L)	132 ± 16	121 ± 14	—


**Ethics approval and consent to participate:** This study was approved by the ethical committee of The Second Affiliated Hospital of Harbin Medical University, and consent was obtained from the patients.

### 2.2 Operative technique

The main difference between the regular surgery group and the delayed ligation group was the femoral vein ligation time. A 3 cm long transverse incision exposed the femoral artery and vein in the inguinal region when anesthesia was sufficient. First, we ligated the trunk artery of the limb, blocked the blood vessel with the ligation suture reserved in the main vein at any time, temporarily reserved the vascular sheath to avoid vascular tearing, successively cut off the muscle, fascia, and other soft tissues, and finally ligated and cut off the main vein until osteotomy.

**Figure 2:**
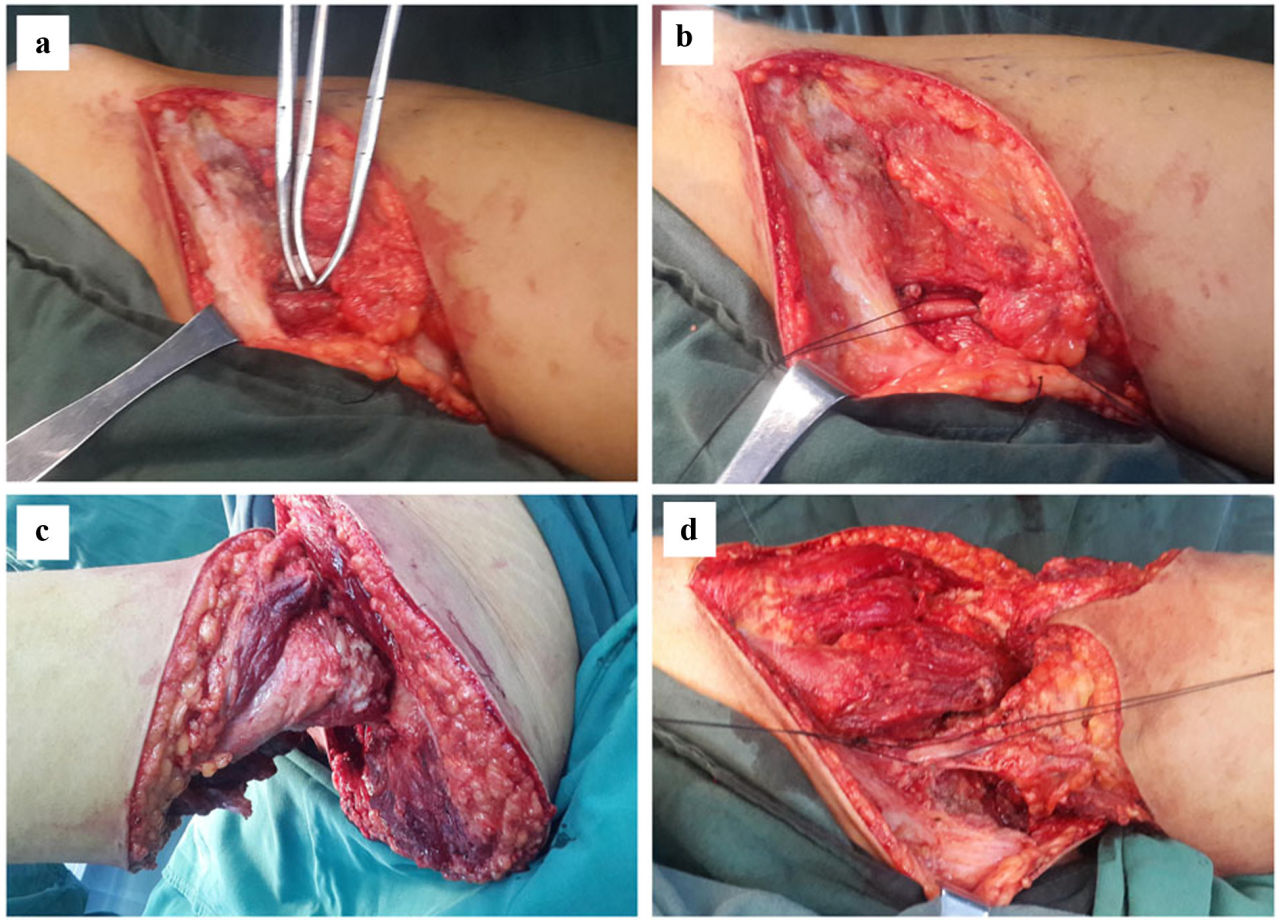
Process of hip disarticulation with delayed femoral vein ligation for the left leg. (a) The exposed main artery was occluded first using a double ligation technique. (b) Both the ligature and sheath of the main vein were reserved. (c) The muscle and fascia were then split, and the backbone and joint capsule of the truncated bone were exposed. (d) Finally, the main vein was ligated, and the distal limb was removed.

The femoral artery and vein were occluded using the double ligation technique in the regular group. Only the artery was occluded in the delayed ligation group (Figure 2a), and both ligature and sheath (Figure 2b) were reserved. In both the regular and delayed ligation groups, the muscle and fascia were split after we expanded the incision. We removed the femoral nerve trunk to expose the backbone and joint capsule of the truncated bone (Figure 2c). Ligation of the main vein was performed in patients of the delayed ligation group. Finally, the distal limb was dissected in both groups (Figure 2d).

### 2.3 Outcome evaluations

The main outcomes in the regular surgery group and the delayed ligation group were mainly evaluated according to operation time and blood loss. The operation time included the time spent on the skin incision, hip disarticulation, and skin suturing. All patients underwent routine blood examinations before and after surgery, respectively. Additionally, blood loss included predicted blood volume (PBV), total blood loss (TBL), hidden blood loss (HBL), intraoperative blood loss, and postoperative blood loss.

PBV was calculated with the following formulas, as described previously [17],

{\text{PBV}}_{\text{male}}=0.3669{H}^{3}+0.03219\hspace{.25em}W+0.6041,]

{\text{PBV}}_{\text{Female}}=0.3561{H}^{3}+0.03308\hspace{.25em}W+0.1833,]

where *H* indicates the height (m) and *W* indicates the weight (kg).

The allowable blood loss was calculated by the following formula as described previously [18]:

{V}_{\text{L}}=\text{PBV}\times ({H}_{\text{O}}-{H}_{\text{F}})/{H}_{\text{AV}}\mathrm{6,041}.]

*H*_O_ indicates the preoperative hematocrit or hemoglobin levels, *H*_F_ indicates the hematocrit or hemoglobin levels postoperatively, and *H*_AV_ indicates the mean value of the difference between pre- and postoperative hematocrit or hemoglobin levels.

TBL and HBL were then calculated as follows:

TBL = *V*_L_ + blood transfusions (1 U packed red blood cells = 200 mL whole blood),

HBL = TBL − (intraoperative blood loss + Postoperative blood loss).

### 2.4 Statistical analysis

All data were analyzed using SPSS software (version 20.0, SPSS Inc., Chicago, IL, USA) and were expressed as mean ± standard deviation with minimum and maximum values. Comparison between the regular surgery group and the delayed ligation group was determined with an independent *t-*test. A *P*-value <0.05 was considered statistically significant.

## 3 Results

### 3.1 Analysis of patient data

A total of 20 patients were included in this study in which 10 patients (28 ± 13 years) were treated using regular surgery, while the other 10 patients (29 ± 15 years) were treated by delayed ligation surgery. Furthermore, in the regular surgery group, the preoperative hemoglobin of patients was 132 ± 16 g/L, while in the delayed ligation group, the preoperative hemoglobin of patients was 121 ± 14 g/L. There are no significant differences in the age, weight, gender, height of patients between the regular surgery group, and the delayed ligation group.

### 3.2 Delayed femoral vein ligation reduced operative time

Operative time is one of the main observation indices for hip disarticulation.

The average operative time of the delayed ligation group was 158 min and that of the regular surgery group was 183 min. As shown in Table 2, the average operation time in the delayed ligation group was significantly shorter than in the regular surgery group (158 ± 23.47 min vs 183 ± 22.63 min, *P* < 0.05).

**Table 2:** The operative time and blood loss of patients who underwent regular surgery or delayed ligation hip disarticulation

Groups	Regular surgery group (*n* = 10)	Delayed ligation group (*n* = 10)	*t*	*P*
Operative time (min)	183 ± 22.63 (161–205 min)	158 ± 23.47 (135–181 min)	2.424	<0.05
TBL (mL)	1357 ± 397.46 (960–1,745 mL)	831 ± 310.30 (521–1,141 mL)	3.302	<0.01
HBL (mL)	852 ± 395.12 (457–1,247 mL)	503 ± 233.72 (270–737 mL)	2.403	<0.05
Intraoperative blood loss (mL)	495 ± 134.26 (361–629 mL)	320 ± 100.55 (220–420 mL)	3.299	<0.01
Postoperative blood loss (mL)	103 ± 34 (69–137 mL)	84 ± 27 (57–111 mL)	1.372	>0.05
PBV (mL)	4,189 ± 855 (3,334–5,044 mL)	4031 ± 680 (3,351–4,711 mL)	0.458	>0.05

PBV: predicted blood volume; TBL: total blood loss; and HBL: hidden blood loss.

### 3.3 Delayed femoral vein ligation decreased blood loss

TBL was in the range of 521–1,141 mL in the delayed ligation group, and the average was 831 mL. In addition, TBL was in the range of 960–1,745 mL in the regular surgery group, and the average was 1,357 mL. TBL decreased significantly in the delayed ligation group compared with that in the regular surgery group (831 ± 310.30 vs 1,357 ± 397.46, *P* < 0.01).

The HBL volume was 270–737.4 mL in the delayed ligation group, and the average HBL volume was 503.7 mL. The HBL volume was 457.6–1247.6 mL in the regular surgery group, and the average HBL volume was 852.6 mL. In the delayed ligation group, the volume of intraoperative blood loss was in the range of 220–420 mL, and the average volume of intraoperative blood loss was 320 mL. In the regular surgery group, the intraoperative blood loss volume was 361–629 mL, and the average volume of intraoperative blood loss was 495 mL. HBL and intraoperative blood loss also decreased significantly in the delayed ligation group than in the regular surgery group (HBL: 503 ± 233.72 mL vs 852 ± 395.12 mL, *P* < 0.05; intraoperative blood loss: 320 ± 100.55 mL vs 495 ± 134.26 mL, *P* < 0.05).

In the delayed ligation group, the volume of postoperative blood loss ranged from 57 to 111 mL, and the average volume of postoperative blood loss was 84 mL. In the regular surgery group, the volume of postoperative blood loss ranged from 69 to 137 mL, and the average volume of postoperative blood loss was 103 mL. However, there were no significant differences in postoperative blood loss and PBV between these two groups (*P* > 0.05).

## 4 Discussion

Hip disarticulation is common for patients with limbs affected by serious trauma, vascular gangrene, infection, and malignant tumors [19]. In hip disarticulation in the cases of malignant tumors, massive dominant and HBLes, as well as long operation times, were considered the most important risk factors for poor outcomes [20,21]. As tumors in the pelvis and thigh have a rich blood supply, temporary or permanent occlusion of the main tumor artery has become an effective method for reducing blood loss and operation time. Some scholars use the method of driving blood by Esmarch’s bandage to reduce the HBL in the nontumor area of the distal limb [22] because of the rich blood supply of the tissue of the proximal limb, the tumor volume, and other factors. Even though the effect of simple distal limb drainage is minor, it is an effective method to reduce HBL, and the author often applied this method at the same time.

Regular techniques are effective for the high-level amputation of lower limbs affected by trauma or gangrene [23,24]. During this process, ligation of major arteries and veins had been considered a standard hemostasis method. However, simultaneous ligation of arteries and veins could lead to a congested distal limb, increase venous pressure, and severe venous distention in part affected by the malignant tumor or lead to massive venous hemorrhage when the soft tissue is split. Meanwhile, extensive blood loss may also be attributable to the removal of the amputated limb. Massive hemorrhage during hip disarticulation not only prolongs the operation time but also threatens the patient’s life. In addition, a previous study indicated that early ligation of adrenal veins is considered a preventive factor to reduce hormonal secretion [25]. By contrast, research reports argue that delayed vein ligation is safer than early ligation [26,27]. Our study also found that delayed vein ligation is safer because the tumor moves from the inferior vena cava to the right and the renal vein moves to the left; thus, possible bleeding instances are controlled more effectively.

In this study, a novel hip disarticulation with delayed femoral vein ligation was performed. The main artery was ligated first during this surgery, and the vascular sheath was reserved to avoid vascular tearing. The main vein was amputated and ligated until the backbone, and joint capsule of the truncated bone was exposed. Hip disarticulation with this novel ligation technique may reduce blood loss. In turn, it will reduce operation time based on a clear surgical field and fully visible anatomy. As expected, significantly reduced operative time, TBL, HBL, and intraoperative blood loss were observed in patients who underwent hip disarticulation with delayed vein ligation, indicating that delayed femoral vein ligation effectively reduced hemorrhage. During amputation, intraoperative blood loss can be influenced by various factors, such as the operator’s proficiency, the age and weight of the patients, and the size and location of the limb tumor [28]. Effective control of intraoperative blood loss and reduction of operative time may also be achieved by applying delayed femoral vein ligation in hemipelvectomy. In addition, no significant differences were found in postoperative blood loss between the two groups, indicating that delayed femoral vein ligation mainly affected hidden and intraoperative blood loss cases. Moreover, Zografos et al. [29] also indicated that delayed vein ligation during laparoscopic adrenalectomy for pheochromocytoma is safe and effective. This procedure could reduce blood loss and operation time. Also, blood loss was associated with complications and increased patient mortality rates [30]. In our study, we found that delayed femoral vein ligation could reduce blood loss. Although we did not count the survival rates and complications of all patients, our research results still provide a new direction for improving the success rate of surgery and the survival rate of patients in the future. Additional investigations, research, and implementation of experiments are needed to explore this technology in depth.

This study has some limitations. First of all, the sample size we collected was relatively small, the observation indicators were relatively limited, and many bias factors affected the experiment. In our research, we did not consider possible complications or the use of other drugs that may have interfered with blood clotting. Future experiments need to expand the sample size and consider multiple factors for more in-depth research.

## 5 Conclusion

In conclusion, delayed femoral vein ligation during hip disarticulation could significantly reduce operation time and hidden and intraoperative blood loss. Although this novel ligation technique could reduce the perioperative bleeding risk for hip disarticulation, additional clinical studies on its application were needed.

## Abbreviations

PBV: predicted blood volume
TBL: total blood loss
HBL: hidden blood loss
SD: standard deviation
